# Marine Biocompounds for Neuroprotection—A Review

**DOI:** 10.3390/md18060290

**Published:** 2020-05-31

**Authors:** Adrian Florian Bălașa, Cristina Chircov, Alexandru Mihai Grumezescu

**Affiliations:** 1 Târgu Mureș Emergency Clinical Hospital, “George Emil Palade” University of Medicine, Pharmacy, Science and Technology of Târgu Mureș, RO-540142 Târgu Mureș, Romania; adrian.balasa@yahoo.fr; 2 Faculty of Applied Chemistry and Materials Science, University Politehnica of Bucharest, RO-060042 Bucharest, Romania; cristina.chircov@yahoo.com; 3 Research Institute of the University of Bucharest (ICUB), University of Bucharest, 060101 Bucharest, Romania

**Keywords:** marine biocompounds, neuroprotection, neurodegeneration, Alzheimer’s disease, Parkinson’s disease

## Abstract

While terrestrial organisms are the primary source of natural products, recent years have witnessed a considerable shift towards marine-sourced biocompounds. They have achieved a great scientific interest due to the plethora of compounds with structural and chemical properties generally not found in terrestrial products, exhibiting significant bioactivity ten times higher than terrestrial-sourced molecules. In addition to the antioxidant, anti-thrombotic, anti-coagulant, anti-inflammatory, anti-proliferative, anti-hypertensive, anti-diabetic, and cardio-protection properties, marine-sourced biocompounds have been investigated for their neuroprotective potential. Thus, this review aims to describe the recent findings regarding the neuroprotective effects of the significant marine-sourced biocompounds.

## 1. Introduction

Considering their co-evolution with the associated biological targets, natural products have been favored by scientists for drug discovery and development in the treatment of various human diseases [1,2]. In this context, natural compounds represent the main treatment strategy for 87% of human diseases [3], with 63% of the newly developed drugs being categorized as naturally derived, either modified natural products, unmodified natural products, or synthetic products with a natural compound as the pharmacophore. Moreover, approximately 68% of all anti-infectious drugs, including antibacterial, antiviral, antifungal, and antiparasitic compounds, and 63% of anti-cancer drugs used between 1981 and 2008 were obtained from natural sources [4]. The advancements in the field of natural products are based on their considerable impacts on the pharmaceutical interests and the associated economic activities. Thus, there is a high interest in the discovery of small drug molecules from templates and designs of biologically and chemically diversified natural product pools, the development of novel separation, purification, and characterization techniques and the establishment of test scaffolds [5,6,7]. In this manner, sampling understudied locations of the planet to enhance the knowledge in the biogeography area is fundamental [8].

While terrestrial organisms represent the primary current source for developing natural therapeutics, there has been an increasing interest in focusing on marine organisms [1]. As oceans occupy more than 70% of the earth’s surface, their biodiversity is a source of various types of micro- and macro-organisms offered by different oceanic zones, which makes them an essential reservoir of natural products [4,9,10,11]. The marine environment, through the use of fish and algae, has represented a source of medicines and oils since ancient times [4,12,13]. The increased marine biodiversity is a result of the different conditions in terms of pressure, temperature, salinity, illumination and nutrient levels, and oxygen and ion concentrations that lead to specific adaptations and specializations of the organisms [1,9,14,15]. Organisms can be found at all depths, from planktonic organisms in the upper ocean and fish and marine mammals that inhabit deeper waters, to benthic organisms that can be found throughout the ocean basins, even at the bottom of the Mariana Trench, 10,900 m below the sea level [16]. Considerable efforts in studying natural marine compounds began in 1951 through the isolation of spongothymidine and spongouridine from the sponge *Cryptotethya crypta* Laubenfels, which led to the synthesis of the anti-cancer agent arabinosyl cytosine [10]. With the advances in deep-sea sample collection, scuba diving, and novel techniques for drug development and aquaculture, an essential number of marine-derived compounds have been discovered and applied for various therapies [11,17]. Subsequently, marine organism exploitation started with collecting large creatures, including red algae, sponges, and soft corals. It continued with microorganism exploitation, such as marine bacteria and cyanobacteria and marine fungi that can produce structurally diverse metabolites [10,15].

The continuously growing interest in marine-derived biocompounds can be justified by the structural and chemical properties that are not usually found in terrestrial products, with several bioactive marine-sourced natural molecules exhibiting considerable bioactivity ten times higher than terrestrial-sourced molecules [1,4,14]. Therefore, marine plants, animals, and lower organisms represent a valuable source of biocompounds, with 650 compounds isolated in 2003, and 3000 active molecules out of 13,000 described currently [4,9,18]. In this manner, marine pharmacology is continuously proving its potential in the biomedical field through the biological functions of the most intensively studied biocompounds, i.e., carbohydrates, polyphenols, peptides, proteins, pigments, and essential fatty acids, which include antioxidant, anti-thrombotic, anti-coagulant, anti-inflammatory, anti-proliferative, anti-hypertensive, anti-diabetic, and cardio-protection properties [18]. In this manner, this review aims to highlight the main bioactive compounds currently used in the biomedical field, with a particular emphasis on the neuroprotective effects of these biocompounds.

## 2. Neurodegenerative Disorders and Mechanisms of Neuroprotection

Neurons continuously require high levels of energy for maintaining protein and organelle quality control, rapid molecule delivery in and out of cells, and transferring organelles and other factors throughout the cell. Since they cannot divide, an impairment of the pathways involved in these functions will subsequently lead to neurodegeneration [19]. Neurodegeneration is a complex progressive multifactorial process that leads to the loss and death of neuronal structures in the nervous system [20]. Mainly occurring in the later stages of life, neurodegeneration is associated with the accumulation of insoluble deposits of protein and peptide aggregates and inclusion bodies in different areas of the brain and spinal cord. These deposits generally contain misfolded proteins, molecular chaperones, and ubiquitin, E3 ligases, and proteasome subunits as components of the ubiquitin–proteasome system [19,21]. Neurodegeneration implies additional underlying mechanisms, such as oxidative stress, calcium deregulation, mitochondrial dysfunction, axonal transport deficits, abnormal neuron–glial interactions, neuroinflammation, DNA damage, and aberrant RNA processing [20]. Consequently, such processes gradually overwhelm the self-defense mechanisms, leading to life–death imbalances and culminating in programmed cell death through several death paths, including apoptosis, necrosis, autophagy, and parthanatos [22].

Hence, neurodegenerative disorders are accompanied by structural, chemical, and electrophysiological abnormalities in the brain and spinal cord, causing muscle weakness, poor coordination, seizures, pain, permanent paralysis and loss of sensation, and cognitive performance alterations and dementia. There is a wide range of neurodegenerative diseases that pose major concerns among aging populations worldwide, including Alzheimer’s disease (AD) and Parkinson’s disease (PD) as the most prevalent ones [20,23,24,25,26]. While certain genes give rise to disease-specific protein inclusions, there is common pathobiology that supports the efficiency of similar therapy strategies for various neurodegenerative diseases [19].

AD is the most common neurodegenerative disorder and the predominant form of dementia among the elderly. With approximately 44 million people living with AD or related dementia and nearly 5 million new cases reported annually, the numbers are expected to double by 2030 and triple by 2050 [27,28,29]. AD is characterized by an abnormal accumulation of amyloid-β proteins as amyloid plaques and hyperphosphorylated tau proteins that form intracellular neurofibrillary tangles. These processes consequently lead to synapse dysfunction and loss, inflammatory responses, and neuronal loss and microtubule disassembly, dendritic collapse, and axonal degeneration, respectively [21,27,29,30]. PD is the second most common neurodegenerative disease, with 273 per 100,000 individuals between the ages of 50 and 59, and 2700 per 100,000 individuals between 70 and 79 [28,31]. PD is a chronic disorder characterized by the progressive degeneration of dopaminergic neurons in the substantia nigra pars compacta and the accumulation of cytoplasmic inclusions and α-synuclein-containing Lewy bodies [21,31,32]. Clinically, PD is a multisystem disorder with both neurologic and systemic manifestations, including unilateral rest tremor, bradykinesia, rigidity, and disordered balance, gait, and falls [31,33]. As current treatments only help to relieve some of the physical and mental symptoms, there is no available cure. With continuous health improvements, there is an increased life expectancy worldwide. Consequently, as there is a greater risk for developing an age-related neurodegenerative disease, novel, and efficient strategies to ensure neuroprotection are fundamental [28].

Neuroprotection is a mechanism that aims to counter the process of neurodegeneration and brain malfunctioning through chemical, genetic, biological, physiological, or behavioral interventions, which could affect pathophysiological or compensatory adaptive neural mechanisms [34]. Subsequently, since spontaneous neural regeneration in the central nervous system does not generally occur, and the neuroplasticity mechanism is usually insufficient, additional strategies must be implied [35]. In this regard, a series of biomaterials in the form of nanoparticles or scaffolds have been investigated for their neuroprotective and neuroregenerative capacities [23], including alginate, gelatin, collagen, chitosan, hyaluronic acid, and poly(lactic-co-glycolic acid). Their use as microcarriers for the release of neuroprotective molecules has the potential to satisfy the therapeutic necessity for pharmacological release by reducing the degradation susceptibility and activity decay and, therefore, extending their action [36,37]. Recent years have witnessed a great focus on the discovery of natural substances with neuroprotective potential that could be efficient in the prevention and/or treatment of neurodegenerative disorders [38]. As oxidative stress has been considered to play an essential role in the onset and progression of neurodegeneration, there has been a significant scientific focus on the development of antioxidant compounds for neuroprotection [39]. The importance of the marine environment as a source of pharmaceutical agents targeting the central nervous system has been demonstrated through various studies regarding the neuroprotective and neuroregenerative effects of marine biocompounds. Thus, the following sections focus on describing the characteristics of such biocompounds and the most recent studies regarding their neuroprotection potential. For this review, the biocompounds were selected based on recent studies from 2018–2020, which have identified potential neuroprotective activities either as neuroprotective bioactive compounds themselves or as carriers for the delivery of drugs for neuroprotection.

## 3. Marine Polysaccharides for Neuroprotection

**Figure 1:**
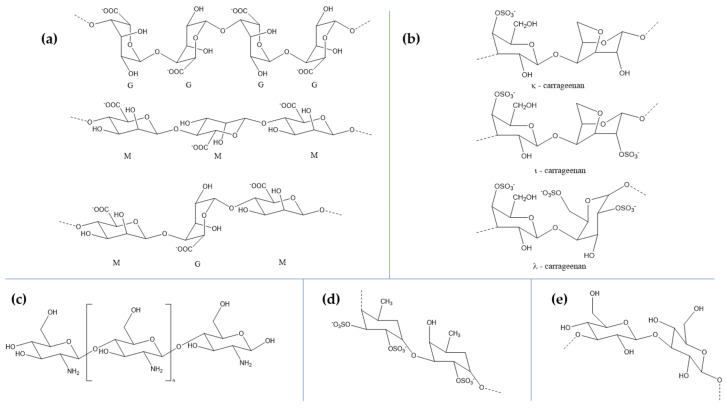
The sources of the main marine-derived polysaccharides: (**a**) alginate; (**b**) carrageenan; (**c**) chitosan; (**d**) fucoidan; (**e**) laminarin

Marine organisms are highly rich in carbohydrates, especially in the form of sulfated and non-sulfated polysaccharides (Table 1, Figure 1) [40]. Chitin is described as a family of polysaccharides composed of linear β-(1,4)-2-acetamido-2-deoxy-D-glucose or N-acetylglucosamine [41,42,43,44]. Chitin, the second most abundant natural polymer of the ecosystem after cellulose [42,44,45,46], represents the main component of the exoskeleton of marine arthropods and crustaceans, especially shrimps, crabs, lobsters, krill, oysters, prawns, and squid [41,42,43,44,45,47]. While it is mainly extracted from the fishing industry waste, some fungi, mollusks, and nematodes can also be a source [43,44,45]. Chitin is obtained as a colorless or off-white powder that is insoluble in aqueous media or polar solvents due to its high cohesive energy caused by strong intermolecular hydrogen bonds formed between amide bonds [41,44,45]. Chitin is mainly used for producing chitosan, which is the partial or full alkaline deacetylation product of chitin [41,43,48,49,50]. In this manner, during the deacetylation process in the presence of sodium hydroxide, the acetyl bonds are broken to form glucosamine [43], and a linear polysaccharide with randomly distributed β-(1,4)-linked D-glucosamine and N-acetyl-D-glucosamine is formed [42,45,49,51,52,53]. Many studies have reported biocompatibility, biodegradability, non-toxicity, immunomodulatory, anti-tumor, antioxidant, hypolipidemic, neuronal regulatory, and anti-microbial properties of chitosan, which highly depend on the degree of deacetylation and the polymer chain size, i.e., an 87 kDa chitosan proved to be more effective than a 532 kDa chitosan against bacterial strains and a lower deacetylation degree leads to a higher degradation rate and host inflammatory response [42,43,48,51,52,54,55]. A wide array of studies investigated the potential of chitosan for neuroprotection. In this manner, chitosan nanoparticles have proved their therapeutic effects on BV-2 glial cells, an immortalized rat microglial line that mimics the characteristics of primary microglia, exposed to hydrogen peroxide [56]. Similar results were obtained using chitooligosaccharides on SH-S5Y5 neurons after exposure to hydrogen peroxide, with the highest activity at the lowest concentration of 0.02 mg/mL [57]. Furthermore, carboxymethylated chitosan protected Schwann cells against hydrogen peroxide-induced damage and apoptosis, resulting in decreased lactate dehydrogenase release and enhanced cell viability through the mitochondrial-dependent pathway [58]. Additionally, similar studies were performed using protocatechuic acid-grafted chitosan and rosmarinic acid-loaded chitosan nanoemulsions on neuron-like rat phaeochromocytoma cells and rat astrocyte primary cultures, respectively. Both studies showed promising neuroprotective effects against hydrogen peroxide and L-glutamic acid-induced apoptosis and LPS-induced oxidative stress [59,60]. Moreover, the neuroprotective effects of chitosan were investigated on PD models. Specifically, low molecular weight sulfated chitosan proved its potential to reduce the consequences of the disease on rotenone-treated SH-SY5Y cells [61]. Similarly, rotigotine- and naringenin-loaded chitosan nanoparticles showed alleviated effects of 6-hydroxydopamine-induced neurotoxicity in SH-SY5Y cells [62,63]. Additionally, rotigotine administration to haloperidol-induced PD rats led to decreased lactate dehydrogenase and increased catalase activities, as well as catalepsy reversal, akinesia, and swimming ability restoration [63]. Beneficial effects have also been shown against multiple sclerosis, using dimethyl fumarate-loaded chitosan nanoparticles on rodent models that led to significantly increased locomotion scores [64]. Another study proved the repair potential of LINGO-1–directed siRNA-loaded chitosan nanoparticles on demyelinated rat models with compromised motor performance and coordination [65]. Additionally, chitosan scaffolds have been shown to give a high nerve fiber regeneration capacity when compared to alginate or chitosan–alginate scaffolds in spinal cord injury rat models [66].

Marine flora has received increasing interest as a source for marine polysaccharides due to their abundance, unique physicochemical properties, and low extraction costs [67]. Marine algae represent an ideal candidate for extracting polysaccharides owing to their various biological properties, including antioxidant, antiviral, antibacterial, anticancer, anti-inflammatory, immunomodulation, blood coagulation, hypolipidemic and hypocholesterolemic, and osteoprotective effects, which are crucial for pharmaceutical and biomedical research [40,67,68,69,70]. They cannot be found elsewhere [71]. The essential seaweed-derived polysaccharides with neuroprotective effects are alginate, carrageenan, fucoidan, and laminarin [67,68,69,71].

Alginate, the term generally used for alginic acid salts and derivatives, but also for alginic acid itself, is a natural linear polysaccharide consisting of β-(1,4)-linked D-mannuronic acid and α-(1,4)-linked L-guluronic acid units [72,73,74,75,76]. Consequently, the polymeric backbone consists of homogenous sequences of mannuronic acid (M) or guluronic acid (G) blocks, and alternating sequences (MG) [72,77,78]. Since β-1,4 linkages confer a 4C1 chair conformation that imparts flexibility to the chain and α-1,4 linkages lead to a 1C4 conformation, which is considerably stiff, the stiffness of the backbone blocks decreases in the order GG, MM, and MG [72]. Alginate is extracted from brown algae, where it exhibits structural functions as a cell wall component, comprising about 30% dry weight. Most common sources of alginate are *Laminaria hyperborea*, *Laminaria digitata*, *Macrocystis pyrifera*, *Ascophyllum nodosum*, and *Laminaria japonica* [72,73,74,75,77,79,80]. Alginate is usually extracted through the dissolution of seaweed biomass with a basic solution, precipitation in calcium chloride, filtration, purification, and drying steps [75,80]. The physicochemical properties of alginate, such as gel formation or viscosity, are directly influenced by the ratio of mannuronic to guluronic acid, the arrangement and length of the blocks, and the molecular size of the polymers, which differ depending on the isolation source or the extraction season [72,75,77]. One research group investigated the neuroprotective effects of seleno-polymannuronate prepared from alginate-derived polymannuronate. Their results suggested that this biocompound exhibited increased mitochondrial membrane potential and inhibition of amyloid-β aggregation and reduced APP and BACE1 protein and cytochrome c expression in N2a-sw cells, proving its potential in preventing neurodegeneration [81]. Moreover, ellagic acid-loaded calcium–alginate nanoparticles were administered to male Swiss albino mice with pentylenetetrazol-induced seizures. Results showed the superior effects of this system, which prevented increased glutamate, decreased γ-aminobutyric acid concentrations, and ameliorated increased amyloid-β and homocysteine levels [82]. Similarly, erythropoietin–alginate microspheres administered in Balb/c-strain mice improved locomotor and glutathione peroxidase activity, with no significant differences when using increased polymer concentrations [83]. Furthermore, paclitaxel-encapsulated poly(lactic-co-glycolic acid) microspheres embedded in alginate hydrogels provided a sustained drug delivery system in spinal cord injury models [84].

Carrageenan comprises a family of hydrophilic high molecular weight biopolymers consisting of linear sulfated galactans [85,86,87,88,89,90]. These galactans are composed of D-galactose and 3,6-anhydrogalactose residues linked through alternating α(1,3) and β(1,4) glycosidic bonds [88,91,92,93]. Carrageenan contains a repeated and alternating structure of 1,3-linked β-d-galactopyranose and 1,4-linked α-d-galactopyranose units [87,94,95]. Depending on their structural features, such as sulfate patterns or 3,6-anhydrogalactose presence on D-galactose units, there are at least 15 distinct types of carrageenans [91,95]. However, owing to their gelling and viscoelastic properties, κ-, ɩ-, and λ-carrageenan are of commercial importance [89,92,93,94,95,96]. Carrageenan is isolated from the extracellular matrix of red edible algae of the Rhodophyceae class [88,89,90,94,95,97]. While the original source was *Chondrus crispus*, the extensive use of carrageenan has led to the introduction of novel species. Specifically, κ-carrageenan and ɩ-carrageenan are produced from warm-water seaweeds, namely *Kappaphycus alverezii* and *Eucheuma denticulatum*, respectively, while cold-water species comprising both haploid and diploid *Chondrus crispus/Gigartina stellate* species produce κ-carrageenan and a mixture of κ-carrageenan and λ-carrageenan, respectively [85,89,98,99]. Carrageenan types form different gels at room temperature, namely κ-carrageenan forms strong and brittle gels, ι-carrageenan forms soft and elastic gels, while λ-carrageenan cannot form gels [93,96,99]. κ-carrageenan isolated from *Hypnea musciformis* red algae exhibited neuroprotective activity in 6-hydroxydopamine-induced neurotoxicity on SH-SY5Y cells by modulating mitochondria transmembrane potential and reducing caspase-3 activity [100].

Fucoidan comprises a complex family of natural water-soluble sulfated polysaccharides [69,95,101,102,103] containing long type I and type II branched chains. Type I chains contain repeating (1,3)-linked α-L-fucopyranose residues, while type II chains contain alternating (1,3)- and (1,4)-linked α-L-fucopyranose residues. Additionally, these compounds also consist of sulfated galactofucans with backbones built of (1,6)-β-D-galacto- and/or (1, 2)-β-D-mannopyranosyl units and other monosaccharides, including uronic acid, xylose, rhamnose, glucose, arabinose, and xylose [69,95,101,102,104]. Fucoidans are mainly isolated from the cell wall and mucous matrix of various species of brown algae, such as mozuku, kombu, limu moui, bladderwrack, and wakame [69,101,102]. Generally, seaweed species, geographic location, and extraction season and procedures directly influence the molecular weight, monosaccharide composition, and sulfate content and position [95,101,105]. Fucoidan has received considerable scientific interest for its neuroprotection activities. In this regard, Ecklonia cava-extracted fucoidan showed significant antioxidant activities on hydrogen peroxide-induced cytotoxicity in PC-12 and MCIXC cells and neuron-protective effects comparable to vitamin C, by regulating mitochondrial function and acetylcholinesterase inhibition [106]. Similarly, the effects induced by the combination of non-invasive low intensity pulsed electric field and fucoidan against hydrogen peroxide-induced neuronal damage were investigated on the motor neuron-like cell line NSC-34, showing improved results and neuroprotective potential [107]. Furthermore, one study proved the protective activity of *Sargassum hemiphyllum*-extracted fucoidan against 6-hydroxydopamine-induced apoptosis on SH-SY5Y cells [108]. One group investigated the neuroprotective effects of five distinct fucoidan types prepared from *Fucus vesiculosus* and *Undaria pinnatifida* against amyloid-β aggregation and cytotoxicity. They demonstrated a wide range of neuroprotective activities that may have the potential to alter amyloid-β neurotoxicity in AD [109]. Additionally, *Laminaria japonica*-extracted fucoidan was investigated for its protective effects on the dopamine system and mitochondrial function of dopaminergic neurons in the rotenone-induced PD rat model. Results showed a significantly reversed nigral dopaminergic neuron and striatal dopaminergic fiber loss, reduced mitochondrial respiratory function as detected by the mitochondrial oxygen consumption, and ameliorated behavioral deficits [110]. Moreover, fucoidan has proved its potential in the treatment of transient global cerebral ischemia in gerbil models by relieving the acceleration and exacerbation of ischemic brain injury through the attenuation of oxidative damage and glial cell activation [111,112].

Laminarin or laminaran is a biodegradable and non-toxic linear polysaccharide consisting of β-D-glucans linked by (1,3) and (1,6) glycosidic bonds at different ratios. It is extracted from the cell wall of brown seaweeds, such as Laminariaceae, the original source, but also from *Laminaria*, *Saccharina*, or *Eisenia* species [67,95]. Laminarin has been investigated for its neuroprotective effects in the Cornu Ammonis 1 field of the hippocampus, which is highly vulnerable to ischemia-reperfusion injury, following transient forebrain ischemia in gerbils using histopathological samples. While pretreatment with 10 mg/kg failed to protect neurons, 50 or 100 mg/kg proved to be efficient as a preventive strategy against injuries following cerebral ischemic insults by attenuating reactive gliosis and reducing pro-inflammatory microglia [113]. While such a dose is relatively high, further studies that focus on elucidating the mechanisms responsible for the neuroprotective action could lead to a decrease in the necessary dose and an enhanced activity.

## 4. Marine Glycosaminoglycans for Neuroprotection

**Figure 2:**
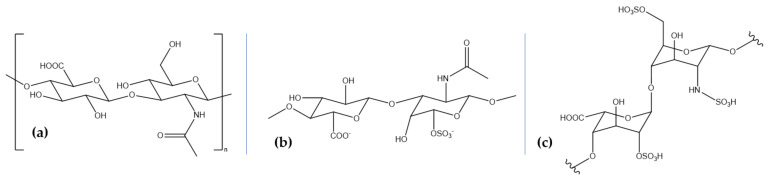
The structure of the main marine-derived glycosaminoglycans: (**a**) hyaluronic acid; (**b**) chondroitin sulfate; (**c**) heparin and heparan sulfate.

Chemically, glycosaminoglycans are long linear heteropolysaccharides consisting of repeating disaccharide units comprising an amino sugar, either N-acetylgalactosamine or N-acetylglucosamine, and uronic acid, either glucuronate or iduronate [114,115,116]. Additionally, sulfate and hydroxyl groups can be present, imparting a strong negative charge and an extended conformation [114]. Excepting hyaluronic acid, glycosaminoglycan chains form proteoglycans by covalently binding to polypeptides as core proteins [114,117]. Although most commercial glycosaminoglycans are extracted from terrestrial animals, marine glycosaminoglycans have achieved a great scientific interest as they are different in terms of molecular weight and sulfation and, consequently, biological activity [118]. Hyaluronic acid (HA), chondroitin sulfate (CS), and heparin and heparan sulfate (HS) (Table 2, Figure 2) are the most physiologically important glycosaminoglycans involved in neuroprotective activities that can be extracted from marine sources.

HA, also known as hyaluronan, is a natural linear, anionic, non-sulfated polysaccharide produced through the polymerization of D-glucuronic acid and N-acetyl-D-glucosamine linked by alternating glucuronidic β-(1,3) or β-(1,4) bonds, activated by the hyaluronan synthase enzyme [89,116,119,120,121,122,123,124]. It provides the backbone for specifically binding domains in the aggregating proteoglycan aggrecan [119,122]. HA is a naturally occurring biopolymer with biocompatible, biodegradable, and viscoelastic properties and a molecular weight ranging from 50 kDa to 2 million kDa [89,123]. As the last decade has witnessed a growing interest in isolating HA from marine organisms, several groups have reported the extraction of low molecular weight HA from the marine bivalves *Mytilus galloprovincialis* and *Amussium pleuronectus* and high molecular weight HA from stingray (*Aetobatus narinari*) liver [125]. Other marine sources include mussels, codfish bones, tuna eyeballs, and shark fins [126]. HA has been investigated for its neuroprotective potential in spinal cord injuries. Specifically, a HA/methylcellulose hydrogel was injected into a syringomyelia rat model, showing improved tissue and functional responses and reduced lipopolysaccharide-mediated microglial activation in vitro [127]. Moreover, this type of hydrogel has been modified with an anti-inflammatory peptide and a brain-derived neurotrophic factor, which significantly enhanced the proliferation of PC12 cells and the recovery in both neurological function and nerve tissue morphology in rat models by regulating inflammatory cytokine levels and improving axonal regeneration [128]. Additionally, HA has also proved its efficiency in stroke management, as neuroglobin-loaded sodium hyaluronate nanoparticles have been intravenously introduced in rat models and reached damaged cerebral parenchyma at early stages [129].

CS is a linear and sulfated polysaccharide consisting of repeating disaccharide units of β-(1,4)-D-glucuronic acid and β-(1,3)-N-acetylgalactosamine, which are usually covalently linked to proteins, forming proteoglycans [130,131,132]. Furthermore, depending on the position of the sulfate group on the polysaccharide backbone, CS can be classified into CS-A (carbon 4), CS-C (carbon 6), CS-E (both carbon 4 and 6), CS-D (position 6 of N-acetylgalactosamine and position 4 of D-glucuronic acid), and CS-B (position 4 of N-acetylgalactosamine and position 2 of D-glucuronic acid) [130,133]. Their versatility has led to a broad range of biological activities and various therapeutic, pharmacological, and nutraceutical applications [134]. Moreover, depending on the sources, either terrestrial or marine, CS contains various chain lengths and oversulfated disaccharides at different relative concentrations, i.e., shark, CS-D, dogfish, CS-A and CS-D, squid and salmon, CS-E, and ray, CS-A and CS-C [133]. Specifically, studies have reported the isolation of CS from the blackmouth catshark (*Galeus melastomus*) [118], corb (*Sciaena umbra*) skin [135], and various shark and other fish cartilage [136]. Experimental researches have indicated the benefits of CS in the therapy of neurodegenerative diseases [137], with one study reporting the neuroprotective effects of CS against advanced glycation end products-induced toxicity, which has been linked to amyloid-β aggregation, oxidative stress, and inflammation [138]. Moreover, low molecular weight CS and selenium-CS nanoparticles have been shown to protect SH-SY5Y cells by inhibiting amyloid-β aggregation, decreasing reactive oxygen species and malondialdehyde levels, and increasing glutathione peroxidase levels [139,140].

Heparin and HS are the most structurally complex glycosaminoglycans, consisting of identical repeating disaccharide units of hexuronic acid, which can be either β-D-glucuronic acid or its C-5 differential isomer, α-L-iduronic acid, and N-acetylgalactosamine through 1,4 linkages. The 2-O position of the hexuronic acid can be sulfated and the 3-O and 6-O positions of the glucosamine can be replaced by an O-sulfo group. The probability of 6-O substitution is greater than 3-O, and the amino group can be sulfonated, acetylated, or unmodified. Heparin and HS have similar structures, but differ in terms of monomer proportion, i.e., heparin has a large proportion of iduronic acid, while HS mainly consists of glucuronic acid. Additionally, heparin has an average of 2.7 sulfated groups compared to 1 in HS, but a lower molecular weight, 12 kDa compared to 30 kDa. Moreover, while heparin is the most negatively charged macromolecule of the human body and, therefore, the most acidic one, HS is characterized by a higher structural heterogeneity [141,142]. The arrangement of the sulfate groups in short segments of the chains produces binding sites for protein ligands to form proteoglycans through O-ether linkages [143,144,145]. Marine animals are evolutionally secluded from terrestrial mammals, and they represent an important source of heparin and HS extraction, as there is a lower risk of microorganism contamination. Thus, marine mollusks, such as bivalves, gastropods, and cephalopods species, sea cucumbers [146], shrimp heads (*Litopenaeus vannamei* and *Penaeus brasiliensis*), clams (*Anomalocardia brasiliana*, *Tivela mactroides*, *Donax striatus*, and *Tapes phlippinarum*), crabs (*Goniopsis cruentata* and *Ucides cordatus*), scallop (*Nodipecten nodosus*), ascidian (*Styela plicata*), sand dollar (*Mellita quinquisperforata*) [147], and cockle (*Cerastoderma edule*) [148] represent great sources of heparin and HS. One study investigated the effects of heparin, heparinase III, chondroitinase, hyaluronic acid, and an MMP-2/9 inhibitor together with amyloid-β oligomers on cortical and hippocampal populations generated from human-induced pluripotent stem cell-derived neural spheroids. Results showed that heparin administration reduces amyloid-β-related neural cell death [149]. Furthermore, heparin administration in gerbils proved that pre-treatment considerably reduces neuronal cell apoptosis and expression of tumor necrosis factor-α and interleukin-1β, thus exerting neuroprotective effects against cerebral ischemia/reperfusion injury [150]. Additionally, HS is a promising therapeutic strategy to protect and repair the brain after stroke, favoring functional recovery [151].

## 5. Marine Glycoproteins for Neuroprotection

Lectins are clusters of oligomeric carbohydrate-binding glycoproteins ubiquitously found in animals, plants, and microorganisms. The “lectin” term is derived from the “legere” Latin word, which means “to select”; precisely, they have a highly specific carbohydrate recognition domain, which offers them the capacity to specifically and reversibly bind to sugar moieties [152,153,154,155,156]. Lectins are a diversified group of proteins of nonimmune origin with many biological roles, including aggregation of animal cells, as they are often referred to as “agglutins”, mediation of cell–cell interactions, homeostatic regulation, and immune recognition of foreign carbohydrates [152,154,155]. Depending on their amino acid sequence and biochemical actions, lectins can be classified in several families. Specifically, lectins in fish include C-type lectins, galectins, pentraxins, X-type lectins/intelectins, calnexin, and calreticulin, which can be found in other animals, and F-type lectins, rhamnose-binding lectins, and pufflectins, which have been discovered in fish. They fulfill different biological roles, such as pathogen recognition and opsonization, complement activation, immune function regulation, or they act as antifreeze proteins or prevent polyspermy during fertilization [155]. So far, lectins have been isolated from a variety of marine animals, such as sponges (*Aplysina lactuca*, *Cliona varians*, *Suberites domuncula*, *Axinella corrugata*, and *Chondrilla caribensis*), annelids (*Cinachyrella apion*, *Chaetopterus variopedatus* and *Serpula vermicularis*), mollusks (*Aplysia dactylomela*, *Mytilus galloprovincialis*, *Argopecten irradians*, *Ruditapes philippinarum*, *Madiolus modiolus*, and *Crenomytilus grayanus*), arthropods (*Tachypleus tridentatus* and *Penaeus monodon*), sea cucumbers (*Holothuria scabra*, *Holothuria grisea*, and *Cucumaria echinata*), ascidians (*Didemnum ternatanum*), amphioxus (*Branchiostoma belcheri* and *Branchiostoma japonicum*), and fish (*Aristichtys nobilis*, *Silurus asotus*, *Oncorhynchus tshawytscha*, *Epinephelus coioides*, *Oncorhynchus mykiss*, *Rachycentron canadum*, and *Paralichthys olivaceus*) [157,158]. As they have the ability to modulate molecular targets in the central nervous system, lectins might be involved in processes associated with neuroplasticity, neurobehavioral effects, and neuroprotection [159]. Therefore, galectin-3, a β-galactoside-binding lectin, could modulate the innate immunity and induce a therapeutic shift in microglia polarization, significantly reducing the infarct size, in the context of ischemic injury [160]. Similarly, pentraxin-3, an angiogenesis key regulator, has demonstrated its potential and clinical relevance by providing sustained long-term neurovascular repair after stroke and reducing neuronal loss [161].

## 6. Marine Lipids and Glycolipids for Neuroprotection

Lipids are responsible for various complex and physiological roles, including cell membrane formation, cell transport, energy storage, signaling, and transmembrane protein modulation. Their composition in the brain depends on age, sex, neuron activity, stress, and trauma, and variations in their concentration, organization, and metabolism might consequently lead to neurological and/or mental disorders [162]. In the brain, the most abundantly found organic compounds are polyunsaturated fatty acids, which are further classified into ω-6 and ω-3 polyunsaturated fatty acids, derived from linoleic acid and α-linolenic acid, respectively [162,163]. While they were generally ignored for more than 40 years, polyunsaturated fatty acids are essential for normal brain development and function [162,163,164,165,166]. ω-3 fatty acids play fundamentally important biological roles, including neurotransmission, signal transduction, receptor binding, and eicosanoid synthesis, and aid in synaptic plasticity and neuroprotection [165,166]. A lack of ω-3 fatty acids has been linked to a chronic pro-inflammatory state in the brain that further leads to dementia and an increased risk of cerebral ischemia [167].

**Figure 3:**
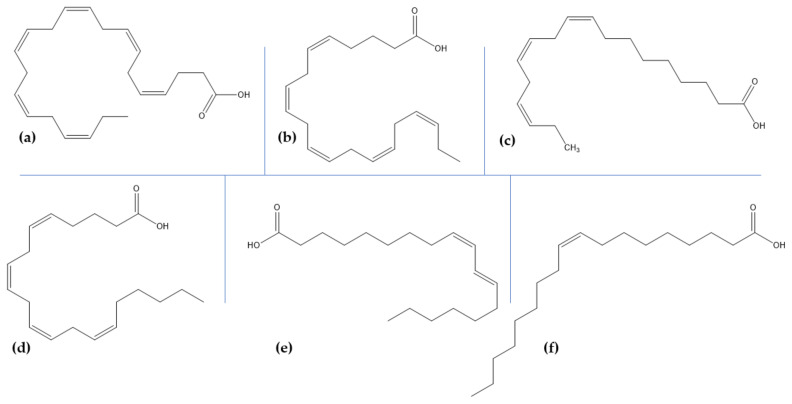
The structure of the main polyunsaturated fatty acids obtained from marine sources: (**a**) docosahexaenoic acid; (**b**) eicosapentaenoic acid; (**c**) α-linolenic acid; (**d**) arachidonic acid; (**e**) linoleic acid; (**f**) oleic acid.

Examples of fatty acids include docosahexaenoic acid, eicosapentaenoic acid, α-linolenic acid, arachidonic acid, linolenic acid, and oleic acid (Table 3, Figure 3) [168]. Fish oils, especially cod liver oil from Atlantic cod (*Gadus Morrhua* L.), are the main source of ω-3 fatty acids [169]. Additionally, microalgae are emerging as a new source for extraction in order to sustain the needs of the population. Due to their increased bioactivity, docosahexaenoic acid, and eicosapentaenoic acid are the most nutritionally significant fatty acids produced [170].

As some studies reported the beneficial effects of some polyunsaturated fatty acids in AD by reducing amyloid-β toxicity through enhancing its degradation and clearance, one group investigated the interactions of fatty acids docosahexaenoic acid, eicosatetraenoic acid, α-linolenic acid, arachidonic acid, linoleic acid, and oleic acid with amyloid-β peptides. Results showed that all the fatty acids tested have anti-aggregation properties by preventing amyloid-β40 and amyloid-β42 fibrillogenesis, thus providing a novel direction for developing a therapy for AD [171]. Furthermore, docosahexaenoic acid has been shown to play a crucial role in neurogenesis, antinociceptive effects, anti -apoptotic effects, synaptic plasticity, Ca^2+^ homeostasis in brain diseases, and nigrostriatal activity functioning, with a high intake of docosahexaenoic acid-containing foods being linked with a lower risk of AD and other brain disorders [172]. In this regard, one study showed that the administration of docosahexaenoic acid-enriched phosphatidylcholine and docosahexaenoic acid-enriched phosphatidylserine in AD SAMP8 mice models improved the metabolic disorders and cognitive deficits by ameliorating amyloid-β pathology, mitochondrial damage, neuroinflammation, and neurotrophic factors and oxidative stress, respectively. The molecular mechanisms responsible have been found to be closely related to the phospholipid polar groups [173]. Moreover, the effects of docosahexaenoic acid and eicosapentaenoic acid, either alone or in combinations of 1:1, 1:2 and 2:1, as found in sea seals, sea algae, and fish oil, on cellular models of AD have been investigated. Results demonstrated that both fatty acids attenuate neuron apoptosis and improve cell viability with synergistic anti-inflammatory effects in the AD model; additionally, pure eicosapentaenoic acid is more effective against oxidative stress, while pure docosahexaenoic acid better improves neurotrophic systems [174]. Furthermore, eicosapentaenoic acid-enriched phospholipids extracted from the sea cucumber (*Cucumaria frondosa*) improved MPTP-induced PD in mice by suppressing oxidative stress and apoptosis and alleviating the loss of dopaminergic neurons via mitochondria-mediated and mitogen-activated protein kinase pathways [175].

Glycolipids comprise a large class of natural compounds consisting of a glycosidic fragment linked to a lipid molecule. Although highly structurally variable, glycolipids can be classified into three main categories, namely glycosphingolipids, glycoglycerolipids, and atypical glycolipids [176].

Chemically, glycosphingolipids comprise sphingosine and fatty acid residues, which are linked to an amide in the ceramide and contain no phosphate groups. Furthermore, a carbohydrate links through a β-glycosidic bond to the primary alcohol oxygen atom of the ceramide [177]. Glycosphingolipids represent the building blocks of the outer leaflet of the cell membrane in a wide variety of terrestrial and marine organisms, where they play fundamental physiological roles due to variations in the sugar chains. They are continuously recycled inside lysosomes by glycosidase fragmentation [178,179]. Based on the constituent sugars, glycosphingolipids can be further classified into cerebrosides, ceramide oligohexosides, globosides, and gangliosides. Echinoderms, porifera, and mollusks have been identified as suitable marine sources for glycosphingolipid isolation [179]. One study investigated the protective effects of sea cucumber-derived cerebrosides against amyloid-β-induced cognitive impairment on AD male rat models. Results proved the neuroprotective capacity of the marine-derived glycolipids by ameliorating neuronal damage and suppressing the induced apoptosis [180]. Moreover, gangliosides, a family of glycosphingolipids containing sialic acid linked to an oligoglycosyl backbone, which is further attached to a ceramide base, are highly expressed in vertebrate nervous systems [181]. Additionally, they are widely present in marine echinoderms, such as sea cucumbers, sea urchins, and starfishes, which are found to be different from mammalian glycosphingolipids in terms of their basic sugar moiety and the types and numbers of sialic acids (i.e., mammalian glycosphingolipids contain mainly N-acetyl-neuraminic acid, while echinoderm glycosphingolipids contain N-acetyl-neuraminic acid, N-glycolylneuraminic acid, and sulfated N-glycolylneuraminic acid). One study reported the neuroprotective effects of the sea urchin *Strongylocentrotus nudus*-isolated glycosphingolipids on AD models of Ab25-35-induced PC12 cells and SAMP8 mice as in vitro and in vivo models. The main mechanisms involve inhibition of synaptic loss through synaptophysin and GAP-43 expression and mediation of mitochondrial apoptosis, which is directly related to the neurofibrillary pathology [182].

Furthermore, glycoglycerolipids are ubiquitously found in the chloroplasts of eukaryotic algae, but also in cyanobacteria or other higher plants. Their basic structure involves a 1,2-diacyl-sn-glycerol moiety and mono- or oligosaccharides attached at the sn-3 position of the glycerol backbone. The three major types of glycoglycerolipids include monogalactosyldiacylglycerol (1,2-diacyl-3-O-(β-D-galactopyranosyl)-sn-glycerol), digalactosyldiacylglycerol 1,2-diacyl-3-O-(α-D-galactopyranosyl-(1′,6)-O-β-D-galactopyranosyl)-sn-glycerol), and sulfoquinovosyldiacylglycerol (1,2-diacyl-3-O-(6-deoxy-6-sulfo-α-D-glucopyranosyl)-sn-glycerol) [179,183]. 

Marine microbial-derived glycolipids have been intensively studied since they are widely produced by a broad spectrum of bacteria extracted from different marine matrices, including animals, e.g., Annelida, *Pteroides spinosum*, or fish gut, and contaminated soils [184]. Furthermore, microalgae have become a promising lipid source due to their lipid accumulation mechanisms triggered by various stress conditions, such as limited nutrients or damaging physical factors [185]. Generally, they produce a great variety of lipids, including polar lipids, neutral lipids, wax esters, hydrocarbons, or sterols [186].

## 7. Marine Pigments for Neuroprotection

Pigments are molecular structures capable of absorbing specific wavelengths of light and reflecting the rest of the visible spectrum. Moreover, microbial pigments possess additional chemical component mixtures that have complex biological activities, such as antimicrobial, anticancer, and immunomodulation. Thus, recent years have witnessed a tremendous increase in the study of terrestrial and marine microbial pigments [187,188,189,190,191]. The production of pigments by marine bacteria is presumably mediated by the quorum-sensing mechanism [188]. Most common types of pigment compounds from marine microorganisms include carotenoids, polyunsaturated hydrocarbons with 30, 40, or 50 carbon atoms in one molecule, melanins, polyphenolic pigments obtained through hydroxylation, oxidation, and polymerization reactions of phenolic compounds, phenazines, tricyclic, redox-active, and small nitrogen-containing heterocyclic aromatic compounds, prodiginines, aromatic chemical compounds with pyrrolyl dipyrromethene core structures, quinones, compounds containing aromatic ring structures with yellow-to-red color hues, tambjamines, alkaloid compounds with yellow color hues, and violacein, indole-pigmented compounds derived from the metabolism of tryptophan [188].

Carotenoids, the most abundant naturally occurring pigments, have received a great scientific interest owing to their potentially beneficial uses in healthcare, pharmaceuticals, and biotechnologies [39,192]. Since the structural elucidation of β-carotene in 1930, about 750 natural carotenoids have been described, among which more than 250 are of marine origin [193]. Carotenoids are generally divided into carotenes, strict hydrocarbon carotenoids with no substituent in their structures, and xanthophylls, oxygen-containing molecules [194]. Among them, the most common carotenoids produced by marine microorganisms, e.g., microalgae, bacteria, archaea, fungi, and fungi-like protists, are β-carotene, astaxanthin, canthaxanthin, β-cryptoxanthin, diadinoxanthin, dinoxanthin, echinenone, fucoxanthin, lycopene, lutein, zeaxanthin, violaxanthin, and rare carotenoids, including bacterioruberin, myxol, salinixanthin, saproxanthin, sioxanthin, and siphonaxanthin. Their extraction is performed by controlling and optimizing the conditions of growth, using fast and low cost techniques [192,193,194].

On the one hand, lycopene is a biocompound that has been widely researched owing to its beneficial effects in the central nervous system. One study proved its potential to attenuate oxidative stress and reduce tert-butyl hydroperoxide-induced cell apoptosis as key factors in the pathogenesis of AD. Lycopene administration led to improved cell viability and neuron morphology, increased GSH/GSSG levels, restored mitochondrial membrane potential, and decreased reactive oxygen species [195]. Similarly, intragastric pretreatment resulted in reduced inflammatory cytokine levels and reversed amyloid-β-induced up-regulation of TLR4 and NF-κB p65 mRNA and protein expressions at the choroid plexus, thereby diminishing amyloid-β deposition in the hippocampus [196]. Additionally, the administration of lycopene has led to alleviated cognition impairment and oxidative stress by decreasing malondialdehyde and 8-hydroxy-2′-deoxyguanosine levels and increasing glutathione level and superoxide dismutase activity in aluminum chloride-induced hippocampal lesions in rat models. These mechanisms have proved to subsequently prevent neuroinflammation and apoptosis [197]. Furthermore, lycopene exhibited neuroprotective effects in MPTP-treated PD mice models by increasing dopamine levels and decreasing oxidative stress levels [198]. Lycopene has also proved to be useful in spinal cord ischemia/reperfusion injury rat models, by improving neurological function recovery and suppressing neuronal death and neuroinflammation [199] and hyperlipidemia-induced cerebral vessel injury prevention by decreasing astrocytes activation and inflammatory cytokine production [200].

On the other hand, astaxanthin, a xanthophyll carotenoid compound, has proved its neuroprotective potential through the inhibition of lipopolysaccharide-induced neuroinflammation, amyloidogenesis, and oxidant activity in mice models [201] and the prevention of hippocampal insulin resistance and AD complications in Wistar rats [202] and brain damage in offspring exposed to prenatal epilepsy seizures [203]. Additionally, astaxanthin and fucoxanthin have also been investigated for their neuroprotective potential against amyloid-β-mediated toxicity in pheochromocytoma neuronal cells. Results demonstrated multi-neuroprotective effects but suggested a higher potential of fucoxanthin as a potential therapeutic strategy [204]. Crocin has also been administered for the therapy of AD and PD, with results proving the potential to treat neurodegeneration [205,206,207]. Additionally, the potential of β-carotene for the treatment of acute spinal cord injury has been investigated, and results showed a reduced progression of secondary injury events through the prevention of the nuclear factor–κB pathway [208].

## 8. Conclusions

As natural products are preferred for the discovery and development of drug molecules for the treatment of various human diseases, there have been considerable advancements in the pharmaceutical biocompound industry. Although terrestrial organisms are currently the main source, the marine environment has received significant scientific interest due to its biodiversity, abundancy, and the biological potential of the derived biocompounds. Additionally, their structural and chemical properties are not generally found in terrestrial products, exhibiting considerable bioactivity ten times higher than terrestrial-sourced molecules. Marine biocompounds are of animal, plant, and microorganism origin, each type providing a plethora of compounds, including carbohydrates, polyphenols, peptides, proteins, pigments, and essential fatty acids, that exhibit antioxidant, anti-thrombotic, anti-coagulant, anti-inflammatory, anti-proliferative, anti-hypertensive, anti-diabetic, and cardio-protection properties. Moreover, marine biocompounds have proved their neuroprotective effects through various research studies, mainly aiming at the prevention of neurodegeneration and the reduction of oxidative stress in the central nervous system. However, the field of marine-sourced neuroprotective compounds is still in its infancy, requiring further discoveries and investigations.

## Figures and Tables

**Table 1:** The sources of the main marine-derived polysaccharides.

Biocompound	Marine Sources
Chitosan	shrimps, crabs, lobsters, krill, oysters, prawns, and squid
Alginate	brown algae (*Laminaria hyperborea*, *Laminaria digitata*, *Macrocystis pyrifera*, *Ascophyllum nodosum*, and *Laminaria japonica*)
Carrageenan	red edible algae of the *Rhodophyceae* class (*Chondrus crispus*, *Kappaphycus alverezii*, *Eucheuma denticulatum*, and *Gigartina stellate*)
Fucoidan	brown algae (mozuku, kombu, limu moui, bladderwrack, and wakame)
Laminarin	brown seaweeds (*Laminariaceae*, *Laminaria*, *Saccharina*, and *Eisenia* species)

**Table 2:** The sources of the main marine-derived glycosaminoglycans.

Biocompound	Marine Sources
Hyaluronic acid	marine bivalves (*Mytilus galloprovincialis* and *Amussium pleuronectus*), stingray (*Aetobatus narinari*), mussels, codfish bones, tuna eyeballs, and shark fins
Chondroitin sulfate	blackmouth catshark (*Galeus melastomus*), corb (*Sciaena umbra*), and shark and fish cartilage
Heparin and heparan sulfate	mollusks (bivalves, gastropods, and cephalopods species), sea cucumbers, shrimp heads (*Litopenaeus vannamei* and *Penaeus brasiliensis*), clams (*Anomalocardia brasiliana*, *Tivela mactroides*, *Donax striatus*, and *Tapes phlippinarum*), crabs (*Goniopsis cruentata* and *Ucides cordatus*), scallop (*Nodipecten nodosus*), ascidian (*Styela plicata*), sand dollar (*Mellita quinquisperforata*), and cockle (*Cerastoderma edule*)

**Table 3:** The sources of the main polyunsaturated fatty acids obtained from marine sources.

Biocompound	Marine Sources
docosahexaenoic acid	fish oils, marine algae, sea cucumber, microalgae
eicosapentaenoic acid	
α-linolenic acid	
arachidonic acid	
linoleic acid	
oleic acid	

## References

[1] Gong H., Luo Z., Chen W., Feng Z.-P., Wang G.-L., Sun H.-S. (2018). Marine Compound Xyloketal B as a Potential Drug Development Target for Neuroprotection. Marine Drugs.

[2] Amer M.S., Barakat K.M., Hassanein A.E.A. (2019). Phthalate derivatives from marine Penicillium decumbens and its synergetic effect against sepsis bacteria. Biointerface Res. Appl. Chem..

[3] Khalifa S.A.M., Elias N., Farag M.A., Chen L., Saeed A., Hegazy M.-E.F., Moustafa M.S., Abd El-Wahed A., Al-Mousawi S.M., Musharraf S.G. (2019). Marine Natural Products: A Source of Novel Anticancer Drugs. Mar. Drugs.

[4] Malve H. (2016). Exploring the ocean for new drug developments: Marine pharmacology. J. Pharm. Bioallied Sci..

[5] Khan R.A. (2018). Natural products chemistry: The emerging trends and prospective goals. Saudi Pharm. J..

[6] Dias-Souza M.V., Dias C.G., Ferreira-Marçal P.H. (2018). Interactions of natural products and antimicrobial drugs: Investigations of a dark matter in chemistry. Biointerface Res. Appl. Chem..

[7] Manciu F.S., Ciubuc J.D., Ochoa K., Dacha P., Subedi M., Guerrero J., Eastman M., Hodges D.R., Bennet K.E. (2019). Comparative spectroscopic analysis of nordihydroguaiaretic acid and related natural products to inhibition of calcium oxalate calculi. Biointerface Res. Appl. Chem..

[8] Moss N.A., Leao T., Glukhov E., Gerwick L., Gerwick W.H., Moore B.S. (2018). Chapter One—Collection, Culturing, and Genome Analyses of Tropical Marine Filamentous Benthic Cyanobacteria. Methods in Enzymology.

[9] Hamed I., Özogul F., Özogul Y., Regenstein J.M. (2015). Marine Bioactive Compounds and Their Health Benefits: A Review. Compr. Rev. Food Sci. Food Saf..

[10] Barbosa M., Valentão P., Andrade P. (2014). Bioactive Compounds from Macroalgae in the New Millennium: Implications for Neurodegenerative Diseases. Mar. Drugs.

[11] Figuerola B., Avila C. (2019). The Phylum Bryozoa as a Promising Source of Anticancer Drugs. Mar. Drugs.

[12] Kosanic M., Rankovic B., Stanojkovic T. (2018). Evaluation of antioxidant, antimicrobial and anticancer effects of three selected marine macroalgae. Rom. Biotechnol. Lett..

[13] Sirakov I., Velichkova K., Rusenova N., Dinev T. (2019). In vitro test of inhibition effect of extracts from three seaweed species distributed at Black sea on different pathogens potentially dangerous for aquaponics. Rom. Biotechnol. Lett..

[14] Carson M.A., Clarke S.A. (2018). Bioactive Compounds from Marine Organisms: Potential for Bone Growth and Healing. Mar. drugs.

[15] Martins A., Vieira H., Gaspar H., Santos S. (2014). Marketed Marine Natural Products in the Pharmaceutical and Cosmeceutical Industries: Tips for Success. Mar. Drugs.

[16] Kirk Cochran J. (2014). Biological Oceanography. Reference Module in Earth Systems and Environmental Sciences.

[17] Blunt J.W., Carroll A.R., Copp B.R., Davis R.A., Keyzers R.A., Prinsep M.R. (2018). Marine natural products. Nat. Prod. Rep..

[18] Suleria H.A.R., Gobe G., Masci P., Osborne S.A. (2016). Marine bioactive compounds and health promoting perspectives; innovation pathways for drug discovery. Trends Food Sci. Technol..

[19] Gan L., Cookson M.R., Petrucelli L., La Spada A.R. (2018). Converging pathways in neurodegeneration, from genetics to mechanisms. Nat. Neurosci..

[20] Farooqui A.A., Farooqui A.A. (2018). Chapter 1—Classification and Molecular Aspects of Neurotraumatic Diseases: Similarities and Differences With Neurodegenerative and Neuropsychiatric Diseases. Ischemic and Traumatic Brain and Spinal Cord Injuries.

[21] Lindholm D., Hyrskyluoto A., Bruelle C., Putkonen N., Korhonen L. (2015). Proteasome Role in Neurodegeneration. Reference Module in Biomedical Sciences.

[22] Fan J., Dawson T.M., Dawson V.L., Beart P., Robinson M., Rattray M., Maragakis N.J. (2017). Cell Death Mechanisms of Neurodegeneration. Neurodegenerative Diseases: Pathology, Mechanisms, and Potential Therapeutic Targets.

[23] Liu Y., Hsu S.-H. (2020). Biomaterials and neural regeneration. Neural Regen. Res..

[24] Madore C., Yin Z., Leibowitz J., Butovsky O. (2020). Microglia, Lifestyle Stress, and Neurodegeneration. Immunity.

[25] Sánchez-López E., Marina M.L., Poole C.F. (2018). Chapter 20—Neuroscience Applications of Capillary Electrophoretic Methods. Capillary Electromigration Separation Methods.

[26] Sardoiwala M.N., Kaundal B., Roy Choudhury S., Hussain C.M. (2018). Chapter 37—Development of Engineered Nanoparticles Expediting Diagnostic and Therapeutic Applications Across Blood–Brain Barrier. Handbook of Nanomaterials for Industrial Applications.

[27] Brahmachari G. (2019). Chapter 1—Discovery and development of anti-inflammatory agents from natural products: An overview. Discovery and Development of Anti-Inflammatory Agents from Natural Products, Brahmachari, G., Ed..

[28] Huang M., Gu X., Gao X., Gao H., Gao X. (2019). 13—Nanotherapeutic strategies for the treatment of neurodegenerative diseases. Brain Targeted Drug Delivery System.

[29] Anitha A., Thanseem I., Vasu M.M., Viswambharan V., Poovathinal S.A., Makowski G.S. (2019). Chapter Three—Telomeres in neurological disorders. Advances in Clinical Chemistry.

[30] Magalingam K.B., Radhakrishnan A., Ping N.S., Haleagrahara N. (2018). Current Concepts of Neurodegenerative Mechanisms in Alzheimer’s Disease. BioMed. Res. Int..

[31] Sharma N., Frontera W.R., Silver J.K., Rizzo T.D. (2020). Chapter 142—Parkinson Disease. Essentials of Physical Medicine and Rehabilitation.

[32] Niethammer M., Eidelberg D., Politis M. (2019). Chapter Five—Network Imaging in Parkinsonian and Other Movement Disorders: Network Dysfunction and Clinical Correlates. International Review of Neurobiology.

[33] Kim S.D., Allen N.E., Canning C.G., Fung V.S.C., Day B.L., Lord S.R. (2018). Chapter 11—Parkinson disease. Handbook of Clinical Neurology.

[34] Gozes I., Levine J., Gozes I., Levine J. (2020). Introduction. Neuroprotection in Autism, Schizophrenia and Alzheimer’s Disease.

[35] Teleanu R.I., Gherasim O., Gherasim T.G., Grumezescu V., Grumezescu A.M., Teleanu D.M. (2019). Nanomaterial-Based Approaches for Neural Regeneration. Pharmaceutics.

[36] Gonzalez Nieto D., Fernández-Serra R., Pérez-Rigueiro J., Panetsos F., Martinez-Murillo R., Guinea G. (2020). Biomaterials to Neuroprotect the Stroke Brain: A Large Opportunity for Narrow Time Windows. Cells.

[37] Fernandez-Serra R., Gallego R., Lozano P., Gonzalez-Nieto D. (2020). Hydrogels for neuroprotection and functional rewiring: A new era for brain engineering. Neural Regen. Res..

[38] Wasik A., Antkiewicz-Michaluk L. (2017). The mechanism of neuroprotective action of natural compounds. Pharmacol. Rep.: PR.

[39] Teleanu R.I., Chircov C., Grumezescu A.M., Volceanov A., Teleanu D.M. (2019). Antioxidant Therapies for Neuroprotection—A Review. J. Clin. Med..

[40] Ruocco N., Costantini S., Guariniello S., Costantini M. (2016). Polysaccharides from the marine environment with pharmacological, cosmeceutical and nutraceutical potential. Molecules.

[41] Shang Q., Jiang H., Cai C., Hao J., Li G., Yu G. (2017). Gut microbiota fermentation of marine polysaccharides and its effects on intestinal ecology: An overview. Carbohydr. Polym..

[42] Tang Y., Cui Y., De Agostini A., Zhang L., Zhang L. (2019). Chapter Eighteen—Biological mechanisms of glycan- and glycosaminoglycan-based nutraceuticals. Progress in Molecular Biology and Translational Science.

[43] Loureiro dos Santos L.A. (2017). Natural Polymeric Biomaterials: Processing and Properties. Reference Module in Materials Science and Materials Engineering.

[44] Deshmukh K., Basheer Ahamed M., Deshmukh R.R., Khadheer Pasha S.K., Bhagat P.R., Chidambaram K., Sadasivuni K.K., Ponnamma D., Kim J., Cabibihan J.J., AlMaadeed M.A. (2017). 3—Biopolymer Composites With High Dielectric Performance: Interface Engineering. Biopolymer Composites in Electronics.

[45] Cardoso M.J., Costa R.R., Mano J.F. (2016). Marine Origin Polysaccharides in Drug Delivery Systems. Mar. drugs.

[46] Rodriguez-Chanfrau J.E., Rodriguez-Riera Z., Gamiotea-Turro D. (2019). Trimethylchitosan hydrochloride obtained from lobster carapace chitin on a bench scale. Biointerface Res. Appl. Chem..

[47] Blanco A., Blanco G., Blanco A., Blanco G. (2017). Chapter 4—Carbohydrates. Medical Biochemistry.

[48] Sánchez-Machado D.I., López-Cervantes J., Correa-Murrieta M.A., Sánchez-Duarte R.G., Cruz-Flores P., de la Mora-López G.S., Nabavi S.M., Silva A.S. (2019). Chapter 4.2—Chitosan. Nonvitamin and Nonmineral Nutritional Supplements.

[49] Alamgir A. (2018). Bioactive Compounds and Pharmaceutical Excipients Derived from Animals, Marine Organisms, Microorganisms, Minerals, Synthesized Compounds, and Pharmaceutical Drugs.

[50] Amanzadi B., Mirzaei E., Hassanzadeh G., Mahdaviani P., Boroumand S., Abdollahi M., Hosseinabdolghaffari A., Majidi R.F. (2019). Chitosan-based layered nanofibers loaded with herbal extract as wound-dressing materials on wound model studies. Biointerface Res. Appl. Chem..

[51] Das B., Patra S. (2017). Chapter 1—Antimicrobials: Meeting the Challenges of Antibiotic Resistance Through Nanotechnology. Nanostructures for Antimicrobial Therapy, Ficai, A., Grumezescu, A.M., Eds..

[52] Faust H.J., Guo Q., Elisseeff J.H., Atala A., Lanza R., Mikos A.G., Nerem R. (2019). Chapter 53—Cartilage Tissue Engineering. Principles of Regenerative Medicine.

[53] Ezzat H.A., Hegazy M.A., Nada N.A., Ibrahim M.A. (2019). Effect of nano metal oxides on the electronic properties of cellulose, chitosan and sodium alginate. Biointerface Res. Appl. Chem..

[54] Li Y., Ju D. (2017). Chapter 12—The Application, Neurotoxicity, and Related Mechanism of Cationic Polymers. Neurotoxicity of Nanomaterials and Nanomedicine, Jiang, X., Gao, H., Eds..

[55] Teixeira M.d.C., Santini A., Souto E.B., Ficai A., Grumezescu A.M. (2017). Chapter 8—Delivery of Antimicrobials by Chitosan-Composed Therapeutic Nanostructures. Nanostructures for Antimicrobial Therapy.

[56] Chen B., Li J., Borgens R.B. (2018). Neuroprotection by chitosan nanoparticles in oxidative stress-mediated injury. BMC Res. Notes.

[57] Santos-Moriano P., Fernandez-Arrojo L., Mengibar M., Belmonte-Reche E., Peñalver P., Acosta F.N., Ballesteros A.O., Morales J.C., Kidibule P., Fernandez-Lobato M. (2018). Enzymatic production of fully deacetylated chitooligosaccharides and their neuroprotective and anti-inflammatory properties. Biocatal. Biotransformation.

[58] He B., Wu F., Fan L., Li X.H., Liu Y., Liu Y.J., Ding W.J., Deng M., Zhou Y. (2018). Carboxymethylated chitosan protects Schwann cells against hydrogen peroxide-induced apoptosis by inhibiting oxidative stress and mitochondria dependent pathway. Eur. J. Pharmacol..

[59] Xu C., Guan S., Wang B., Wang S., Wang Y., Sun C., Ma X., Liu T. (2018). Synthesis of protocatechuic acid grafted chitosan copolymer: Structure characterization and in vitro neuroprotective potential. Int. J. Biol. Macromol..

[60] Fachel F.N.S., Dal Prá M., Azambuja J.H., Endres M., Bassani V.L., Koester L.S., Henriques A.T., Barschak A.G., Teixeira H.F., Braganhol E. (2020). Glioprotective Effect of Chitosan-Coated Rosmarinic Acid Nanoemulsions Against Lipopolysaccharide-Induced Inflammation and Oxidative Stress in Rat Astrocyte Primary Cultures. Cell. Mol. Neurobiol..

[61] Manigandan V., Nataraj J., Karthik R., Manivasagam T., Saravanan R., Thenmozhi A.J., Essa M.M., Guillemin G.J. (2019). Low Molecular Weight Sulfated Chitosan: Neuroprotective Effect on Rotenone-Induced In Vitro Parkinson’s Disease. Neurotox. Res..

[62] Md S., Alhakamy N.A., Aldawsari H.M., Asfour H.Z. (2019). Neuroprotective and antioxidant effect of naringenin-loaded nanoparticles for nose-to-brain delivery. Brain Sci..

[63] Bhattamisra S.K., Shak A.T., Xi L.W., Safian N.H., Choudhury H., Lim W.M., Shahzad N., Alhakamy N.A., Anwer M.K., Radhakrishnan A.K. (2020). Nose to brain delivery of rotigotine loaded chitosan nanoparticles in human SH-SY5Y neuroblastoma cells and animal model of Parkinson‘s disease. Int. J. Pharm..

[64] Smriti O., Babita K., Hina C. (2019). Neuroprotective potential of dimethyl fumarate-loaded polymeric Nanoparticles against multiple sclerosis. Indian J. Pharm. Sci..

[65] Youssef A.E.H., Dief A.E., El Azhary N.M., Abdelmonsif D.A., El-fetiany O.S. (2019). LINGO-1 siRNA nanoparticles promote central remyelination in ethidium bromide-induced demyelination in rats. J. Physiol. Biochem..

[66] Yao Z.A., Chen F.J., Cui H.L., Lin T., Guo N., Wu H.G. (2018). Efficacy of chitosan and sodium alginate scaffolds for repair of spinal cord injury in rats. Neural Regen. Res..

[67] Zargarzadeh M., Amaral A.J.R., Custódio C.A., Mano J.F. (2020). Biomedical applications of laminarin. Carbohydr. Polym..

[68] Patel S., Qin Y. (2018). 4—Seaweed-Derived Sulfated Polysaccharides: Scopes and Challenges in Implication in Health Care. Bioactive Seaweeds for Food Applications.

[69] Shen P., Yin Z., Qu G., Wang C., Qin Y. (2018). 11—Fucoidan and Its Health Benefits. Bioactive Seaweeds for Food Applications.

[70] Nechifor R., Nastuneac V., Domingues V.F., Figueiredo S., De Freitas O.M., Delerue-Matos C., Lazar I. (2019). The Use of Marine Algae in the Bioremediation of Contaminated Water with Pharmaceutical Products and Persistent Organic Products (POPs). Rom. Biotechnol. Lett..

[71] Anyanwu R.C., Rodriguez C., Durrant A., Olabi A.G. (2018). Micro-Macroalgae Properties and Applications. Reference Module in Materials Science and Materials Engineering.

[72] Alba K., Kontogiorgos V., Melton L., Shahidi F., Varelis P. (2019). Seaweed Polysaccharides (Agar, Alginate Carrageenan). Encyclopedia of Food Chemistry.

[73] Alihosseini F., Sun G. (2016). 10—Plant-based compounds for antimicrobial textiles. Antimicrobial Textiles.

[74] Tariverdian T., Navaei T., Milan P.B., Samadikuchaksaraei A., Mozafari M., Mozafari M., Singh Chauhan N.P. (2019). Chapter 16—Functionalized polymers for tissue engineering and regenerative medicines. Advanced Functional Polymers for Biomedical Applications.

[75] Abhilash M., Thomas D., Sadasivuni K.K., Ponnamma D., Kim J., Cabibihan J.J., AlMaadeed M.A. (2017). 15—Biopolymers for Biocomposites and Chemical Sensor Applications. Biopolymer Composites in Electronics.

[76] Abdelghany A.M., Meikhail M.S., El-Bana A.A. (2019). Microbial activity and swelling behavior of chitosan/polyvinyl alcohol/sodium alginate semi-natural terpolymer interface containing amoxicillin for wound dressing applications. Biointerface Res. Appl. Chem..

[77] Takeshita S., Oda T., Kim S.-K., Toldrá F. (2016). Chapter Seven—Usefulness of Alginate Lyases Derived from Marine Organisms for the Preparation of Alginate Oligomers with Various Bioactivities. Advances in Food and Nutrition Research.

[78] Azeem M., Batool F., Iqbal N., Ikram ul H., Zia K.M., Zuber M., Ali M. (2017). Chapter 1—Algal-Based Biopolymers. Algae Based Polymers, Blends, and Composites.

[79] Nesic A.R., Seslija S.I. (2017). 19—The influence of nanofillers on physical–chemical properties of polysaccharide-based film intended for food packaging. Food Packaging, Grumezescu, A.M., Ed..

[80] Qin Y., Jiang J., Zhao L., Zhang J., Wang F., Grumezescu A.M., Holban A.M. (2018). Chapter 13—Applications of Alginate as a Functional Food Ingredient. Biopolymers for Food Design.

[81] Bi D., Li X., Li T., Li X., Lin Z., Yao L., Li H., Xu H., Hu Z., Zhang Z. (2020). Characterization and Neuroprotection Potential of Seleno-Polymannuronate. Front. Pharmacol..

[82] El-Missiry M.A., Othman A.I., Amer M.A., Sedki M., Ali S.M., El-Sherbiny I.M. (2020). Nanoformulated ellagic acid ameliorates pentylenetetrazol-induced experimental epileptic seizures by modulating oxidative stress, inflammatory cytokines and apoptosis in the brains of male mice. Metab. Brain Dis..

[83] Hariyadi D.M., Rahmadi M., Rahman Z. (2018). In vivo neuroprotective activity of erythropoietin-alginate microspheres at different polymer concentrations. Asian J. Pharm..

[84] Nazemi Z., Nourbakhsh M.S., Kiani S., Heydari Y., Ashtiani M.K., Daemi H., Baharvand H. (2020). Co-delivery of minocycline and paclitaxel from injectable hydrogel for treatment of spinal cord injury. J. Control. Release.

[85] Barrett B., Rakel D. (2018). Chapter 18—Viral Upper Respiratory Infection. Integrative Medicine.

[86] Jamwal S., Kumar P., Conn P.M. (2017). Chapter 19—Animal Models of Inflammatory Bowel Disease. Animal Models for the Study of Human Disease.

[87] BeMiller J.N. (2019). 13—Carrageenans. Carbohydrate Chemistry for Food Scientists (Third Edition), BeMiller, J.N., Ed..

[88] Suner S.S., Sahiner M., Sengel S.B., Rees D.J., Reed W.F., Sahiner N., Makhlouf A.S.H., Abu-Thabit N.Y. (2018). 17—Responsive biopolymer-based microgels/nanogels for drug delivery applications. Stimuli Responsive Polymeric Nanocarriers for Drug Delivery Applications.

[89] Li R., Wu G., Chen Y. (2020). Chapter 5—Preparation of polysaccharide-based hydrogels via radiation technique. Hydrogels Based on Natural Polymers.

[90] Wang H.-M.D., Li X.-C., Lee D.-J., Chang J.-S. (2017). Potential biomedical applications of marine algae. Bioresour. Technol..

[91] Guedes A.C., Amaro H.M., Sousa-Pinto I., Malcata F.X., Pandey A., Chang J.-S., Soccol C.R., Lee D.-J., Chisti Y. (2019). Chapter 16—Algal spent biomass—A pool of applications. Biofuels from Algae.

[92] Zhang H., Zhang F., Yuan R., Chen Y. (2020). Chapter 13—Applications of natural polymer-based hydrogels in the food industry. Hydrogels Based on Natural Polymers.

[93] Mohanraj R., Grumezescu V., Grumezescu A.M. (2019). Chapter 2—Plant-derived resorbable polymers in tissue engineering. Materials for Biomedical Engineering.

[94] Sudhakar Y.N., Selvakumar M., Bhat D.K., Sudhakar Y.N., Selvakumar M., Bhat D.K. (2018). Chapter 4—Biopolymer Electrolytes for Solar Cells and Electrochemical Cells. Biopolymer Electrolytes.

[95] Shanmugam H., Sathasivam R., Rathinam R., Arunkumar K., Carvalho I.S., Barh D., Azevedo V. (2018). Chapter 3—Algal Biotechnology: An Update From Industrial and Medical Point of View. Omics Technologies and Bio-Engineering.

[96] Zoratto N., Matricardi P., Pal K., Banerjee I. (2018). 4—Semi-IPNs and IPN-based hydrogels. Polymeric Gels.

[97] Qin Y., Qin Y. (2018). 1—Seaweed Bioresources. Bioactive Seaweeds for Food Applications.

[98] Blakemore W.R. (2016). Polysaccharide Ingredients: Carrageenan. Reference Module in Food Science.

[99] Qin Y., Qin Y. (2018). 3—Production of Seaweed-Derived Food Hydrocolloids. Bioactive Seaweeds for Food Applications.

[100] Souza R.B., Frota A.F., Silva J., Alves C., Neugebauer A.Z., Pinteus S., Rodrigues J.A.G., Cordeiro E.M.S., de Almeida R.R., Pedrosa R. (2018). In vitro activities of kappa-carrageenan isolated from red marine alga Hypnea musciformis: Antimicrobial, anticancer and neuroprotective potential. Int. J. Biol. Macromol..

[101] Wang Y., Xing M., Cao Q., Ji A., Liang H., Song S. (2019). Biological Activities of Fucoidan and the Factors Mediating Its Therapeutic Effects: A Review of Recent Studies. Mar. Drugs.

[102] Sang V.T., Ngo D.-H., Kang K.H., Jung W.-K., Kim S.J. (2015). The beneficial properties of marine polysaccharides in alleviation of allergic responses. Mol. Nutr. Food Res..

[103] Gokarneshan N., Rajendran S. (2019). 19—Application of natural polymers and herbal extracts in wound management. Advanced Textiles for Wound Care.

[104] Gao Y., Zhang L., Jiao W., Zhang L. (2019). Chapter Seven—Marine glycan-derived therapeutics in China. Progress in Molecular Biology and Translational Science.

[105] Wang J., Geng L., Yue Y., Zhang Q., Zhang L. (2019). Chapter Six—Use of fucoidan to treat renal diseases: A review of 15 years of clinic studies. Progress in Molecular Biology and Translational Science.

[106] Park S.K., Kang J.Y., Kim J.M., Park S.H., Kwon B.S., Kim G.H., Heo H.J. (2018). Protective effect of fucoidan extract from Ecklonia cava on hydrogen peroxide-induced neurotoxicity. J. Microbiol. Biotechnol..

[107] Hsieh C.H., Lu C.H., Kuo Y.Y., Lin G.B., Chao C.Y. (2019). The protective effect of non-invasive low intensity pulsed electric field and fucoidan in preventing oxidative stress-induced motor neuron death via ROCK/Akt pathway. PLoS ONE.

[108] Huang C.Y., Kuo C.H., Chen P.W. (2018). Compressional-puffing pretreatment enhances neuroprotective effects of fucoidans from the brown seaweed sargassum hemiphyllum on 6-hydroxydopamine-induced apoptosis in SH-SY5Y cells. Molecules.

[109] Alghazwi M., Smid S., Karpiniec S., Zhang W. (2019). Comparative study on neuroprotective activities of fucoidans from Fucus vesiculosus and Undaria pinnatifida. Int. J. Biol. Macromol..

[110] Zhang L., Hao J., Zheng Y., Su R., Liao Y., Gong X., Liu L., Wang X. (2018). Fucoidan protects dopaminergic neurons by enhancing the mitochondrial function in a rotenone-induced rat model of parkinson‘s disease. Aging Dis..

[111] Ahn J.H., Shin M.C., Kim D.W., Kim H., Song M., Lee T.K., Lee J.C., Kim H., Cho J.H., Kim Y.M. (2019). Antioxidant properties of fucoidan alleviate acceleration and exacerbation of hippocampal neuronal death following transient global cerebral ischemia in high-fat diet-induced obese gerbils. Int. J. Mol. Sci..

[112] Kim H., Ahn J.H., Song M., Kim D.W., Lee T.K., Lee J.C., Kim Y.M., Kim J.D., Cho J.H., Hwang I.K. (2019). Pretreated fucoidan confers neuroprotection against transient global cerebral ischemic injury in the gerbil hippocampal CA1 area via reducing of glial cell activation and oxidative stress. Biomed. Pharmacother..

[113] Lee T.K., Ahn J.H., Park C.W., Kim B., Park Y.E., Lee J.C., Park J.H., Yang G.E., Shin M.C., Cho J.H. (2020). Pre-treatment with laminarin protects hippocampal CA1 pyramidal neurons and attenuates reactive gliosis following transient forebrain ischemia in gerbils. Mar. Drugs.

[114] Kumari A., Kumari A. (2018). Chapter 15—Mucopolysaccharidoses. Sweet Biochemistry.

[115] Duan J., Amster I.J., Kanawati B., Schmitt-Kopplin P. (2019). Chapter 20—Application of FTMS to the analysis of glycosaminoglycans. Fundamentals and Applications of Fourier Transform Mass Spectrometry.

[116] Tripathy N., Ahmad R., Song J.E., Khang G., Narayan R. (2019). Biomimetic Approaches for Regenerative Engineering. Encyclopedia of Biomedical Engineering.

[117] Letourneau P.C. (2017). Axonal Pathfinding: Extracellular Matrix Role. Reference Module in Neuroscience and Biobehavioral Psychology.

[118] Vázquez J.A., Fraguas J., Novoa-Carvallal R., Reis R.L., Antelo L.T., Pérez-Martín R.I., Valcarcel J. (2018). Isolation and chemical characterization of chondroitin sulfate from cartilage by-products of blackmouth catshark (Galeus melastomus). Mar. Drugs.

[119] Heinegård D., Lorenzo P., Önnerfjord P., Saxne T., Hochberg M.C., Silman A.J., Smolen J.S., Weinblatt M.E., Weisman M.H. (2015). 5—Articular cartilage. Rheumatology.

[120] Chircov C., Grumezescu A.M., Bejenaru L.E. (2018). Hyaluronic acid-based scaffolds for tissue engineering. Rom. J. Morphol. Embryol..

[121] Silva A.L., Moura L.I.F., Carreira B., Conniot J., Matos A.I., Peres C., Sainz V., Silva L.C., Gaspar R.S., Florindo H.F., Sarmento B., das Neves J. (2018). Chapter 14—Functional Moieties for Intracellular Traffic of Nanomaterials. Biomedical Applications of Functionalized Nanomaterials.

[122] Frisbie D.D., McIlwraith C.W., Frisbie D.D., Kawcak C.E., van Weeren P.R. (2016). 13—Hyaluronan. Joint Disease in the Horse.

[123] Kumar S., Ali J., Baboota S., Maiti S., Jana S. (2019). 16—Polysaccharide nanoconjugates for drug solubilization and targeted delivery. Polysaccharide Carriers for Drug Delivery.

[124] Hu M.-Y., Nukavarapu S., Mozafari M., Sefat F., Atala A. (2019). 11—Scaffolds for cartilage tissue engineering. Handbook of Tissue Engineering Scaffolds: Volume One.

[125] Giji S., Arumugam M., Kim S.-K. (2014). Chapter Four—Isolation and Characterization of Hyaluronic Acid from Marine Organisms. Advances in Food and Nutrition Research.

[126] Abdallah M. (2019). Extraction of hyaluronic acid and chondroitin sulfate from marine biomass for their application in the treatment of the dry eye disease. Acta Ophthalmol..

[127] Ho M.T., Teal C.J., Shoichet M.S. (2019). A hyaluronan/methylcellulose-based hydrogel for local cell and biomolecule delivery to the central nervous system. Brain Res. Bull..

[128] He Z., Zang H., Zhu L., Huang K., Yi T., Zhang S., Cheng S. (2019). An anti-inflammatory peptide and brain-derived neurotrophic factor-modified hyaluronan-methylcellulose hydrogel promotes nerve regeneration in rats with spinal cord injury. Int. J. Nanomed..

[129] Blanco S., Peralta S., Morales M.E., Martínez-Lara E., Pedrajas J.R., Castán H., Peinado M.Á., Ruiz M.A. (2020). Hyaluronate nanoparticles as a delivery system to carry neuroglobin to the brain after stroke. Pharmaceutics.

[130] Nurunnabi M., Revuri V., Huh K.M., Lee Y.-k., Andronescu E., Grumezescu A.M. (2017). Chapter 14—Polysaccharide based nano/microformulation: An effective and versatile oral drug delivery system. Nanostructures for Oral Medicine.

[131] Rimondo S., Perale G., Rossi F., Maiti S., Jana S. (2019). 6—Polysaccharide-based scaffold for tissue-regeneration. Functional Polysaccharides for Biomedical Applications.

[132] Lee S., Oprea A.E., Grumezescu A.M. (2017). Chapter 4—Strategic Design of Delivery Systems for Nutraceuticals. Nanotechnology Applications in Food.

[133] Vázquez J.A., Rodríguez-Amado I., Montemayor M.I., Fraguas J., González M.D.P., Murado M.A. (2013). Chondroitin Sulfate, Hyaluronic Acid and Chitin/Chitosan Production Using Marine Waste Sources: Characteristics, Applications and Eco-Friendly Processes: A Review. Mar. Drugs.

[134] Khwaldia K., Nabavi S.M., Silva A.S. (2019). Chapter 2.3—Chondroitin and Glucosamine. Nonvitamin and Nonmineral Nutritional Supplements.

[135] Bougatef H., Krichen F., Capitani F., Amor I.B., Maccari F., Mantovani V., Galeotti F., Volpi N., Bougatef A., Sila A. (2018). Chondroitin sulfate/dermatan sulfate from corb (Sciaena umbra) skin: Purification, structural analysis and anticoagulant effect. Carbohydr. Polym..

[136] Konovalova I., Novikov V., Kuchina Y., Dolgopiatova N. (2020). Technology and Properties of Chondroitin Sulfate from Marine Hydrobionts. KnE Life Sciences.

[137] Gromova O.A., Torshin I.Y., Semenov V.A., Stakhovskaya L.I., Rudakov K.V. (2019). On the neurological roles of chondroitin sulfate and glucosamine sulfate: A systematic analysis. Nevrol. Neiropsikhiatriya Psikhosomatika.

[138] Iannuzzi C., Borriello M., D’Agostino A., Cimini D., Schiraldi C., Sirangelo I. (2019). Protective effect of extractive and biotechnological chondroitin in insulin amyloid and advanced glycation end product-induced toxicity. J. Cell. Physiol..

[139] Zhang Q., Na Z., Cheng Y., Wang F. (2018). Low-molecular-weight chondroitin sulfate attenuated injury by inhibiting oxidative stress in amyloid β-treated SH-SY5Y cells. NeuroReport.

[140] Gao F., Zhao J., Liu P., Ji D., Zhang L., Zhang M., Li Y., Xiao Y. (2020). Preparation and in vitro evaluation of multi-target-directed selenium-chondroitin sulfate nanoparticles in protecting against the Alzheimer’s disease. Int. J. Biol. Macromol..

[141] Olgierd B., Sklarek A., Siwek P., Waluga E., Łos M.J., Hudecki A., Wiecheć E. (2019). Chapter 11—Methods of Biomaterial-Aided Cell or Drug Delivery: Extracellular Matrix Proteins as Biomaterials. Stem Cells and Biomaterials for Regenerative Medicine.

[142] Xu K., Jin L. (2020). The role of heparin/heparan sulphate in the IFN-γ-led Arena. Biochimie.

[143] Sekiguchi R., Yamada K.M., Litscher E.S., Wassarman P.M. (2018). Chapter Four—Basement Membranes in Development and Disease. Current Topics in Developmental Biology.

[144] Rudd T., Skidmore M.A., Yates E.A., Garg H.G., Linhardt R.J., Hales C.A. (2005). Chapter 12—Surface-Based Studies of Heparin/Heparan Sulfate-Protein Interactions: Considerations for Surface Immobilisation of HS/Heparin Saccharides and Monitoring Their Interactions with Binding Proteins. Chemistry and Biology of Heparin and Heparan Sulfate.

[145] De Pasquale V., Pavone L.M. (2019). Heparan sulfate proteoglycans: The sweet side of development turns sour in mucopolysaccharidoses. Biochim. Biophys. Acta (BBA)—Mol. Basis Dis..

[146] Saravanan R., Kim S.-K. (2014). Chapter Three—Isolation of Low-Molecular-Weight Heparin/Heparan Sulfate from Marine Sources. Advances in Food and Nutrition Research.

[147] Valcarcel J., Novoa-Carballal R., Pérez-Martín R.I., Reis R.L., Vázquez J.A. (2017). Glycosaminoglycans from marine sources as therapeutic agents. Biotechnol. Adv..

[148] Aldairi A.F., Ogundipe O.D., Pye D.A. (2018). Antiproliferative Activity of Glycosaminoglycan-Like Polysaccharides Derived from Marine Molluscs. Mar. Drugs.

[149] Bejoy J., Song L., Wang Z., Sang Q.X., Zhou Y., Li Y. (2018). Neuroprotective Activities of Heparin, Heparinase III, and Hyaluronic Acid on the Aβ42-Treated Forebrain Spheroids Derived from Human Stem Cells. ACS Biomater. Sci. Eng..

[150] Ye Q., Hai K., Liu W., Wang Y., Zhou X., Ye Z., Liu X. (2019). Investigation of the protective effect of heparin pre-treatment on cerebral ischaemia in gerbils. Pharm. Biol..

[151] Khelif Y., Toutain J., Quittet M.S., Chantepie S., Laffray X., Valable S., Divoux D., Sineriz F., Pascolo-Rebouillat E., Papy-Garcia D. (2018). A heparan sulfate-based matrix therapy reduces brain damage and enhances functional recovery following stroke. Theranostics.

[152] Stillwell W., Stillwell W. (2016). Chapter 9—Basic Membrane Properties of the Fluid Mosaic Model. An Introduction to Biological Membranes.

[153] Marutescu L., Popa M., Saviuc C., Lazar V., Chifiriuc M.C., Grumezescu A.M. (2017). 8—Botanical pesticides with virucidal, bactericidal, and fungicidal activity. New Pesticides and Soil Sensors.

[154] Kalač P., Kalač P. (2016). Chapter 4—Health-Stimulating Compounds and Effects. Edible Mushrooms.

[155] Castro R., Tafalla C., Beck B.H., Peatman E. (2015). 2—Overview of fish immunity. Mucosal Health in Aquaculture.

[156] Samoilova N.A., Krayukhina M.A., Popov D.A., Anuchina N.M., Piskarev V.E. (2018). 3’-sialyllactose-decorated silver nanoparticles: Lectin binding and bactericidal properties. Biointerface Res. Appl. Chem..

[157] Randy C., Wong J., Pan W., Chan Y., Yin C., Dan X., Ng T. (2015). Marine lectins and their medicinal applications. Appl. Microbiol. Biotechnol..

[158] Marques D.N., Almeida A.S.d., Sousa A.R.d.O., Pereira R., Andrade A.L., Chaves R.P., Carneiro R.F., Vasconcelos M.A.d., Nascimento-Neto L.G.d., Pinheiro U. (2018). Antibacterial activity of a new lectin isolated from the marine sponge Chondrilla caribensis. Int. J. Biol. Macromol..

[159] Araújo J.R.C., Coelho C.B., Campos A.R., de Azevedo Moreira R., de Oliveira Monteiro-Moreira A.C. (2020). Animal Galectins and Plant Lectins as Tools for Studies in Neurosciences. Curr. Neuropharmacol..

[160] Rahimian R., Lively S., Abdelhamid E., Lalancette-Hebert M., Schlichter L., Sato S., Kriz J. (2019). Delayed Galectin-3-Mediated Reprogramming of Microglia After Stroke is Protective. Mol. Neurobiol..

[161] Rajkovic I., Wong R., Lemarchand E., Rivers-Auty J., Rajkovic O., Garlanda C., Allan S.M., Pinteaux E. (2018). Pentraxin 3 promotes long-term cerebral blood flow recovery, angiogenesis, and neuronal survival after stroke. J. Mol. Med..

[162] Lange K.W. (2020). Omega-3 fatty acids and mental health. Glob. Health J..

[163] McNamara R.K., Asch R.H., Lindquist D.M., Krikorian R. (2018). Role of polyunsaturated fatty acids in human brain structure and function across the lifespan: An update on neuroimaging findings. Prostaglandins Leukot. Essent. Fat. Acids.

[164] Spector A.A., Kim H.-Y. (2019). Emergence of omega-3 fatty acids in biomedical research. Prostaglandins Leukot. Essent. Fat. Acids.

[165] Ouyang W.-C., Sun G.-C., Hsu M.-C. (2020). Omega-3 fatty acids in cause, prevention and management of violence in schizophrenia: Conceptualization and application. Aggress. Violent Behav..

[166] Harrington J.W., Bora S., Rakel D. (2018). Chapter 8—Autism Spectrum Disorder. Integrative Medicine.

[167] Zhang W., Chen R., Yang T., Xu N., Chen J., Gao Y., Stetler R.A. (2018). Fatty acid transporting proteins: Roles in brain development, aging, and stroke. Prostaglandins Leukot. Essent. Fat. Acids.

[168] Gupta C., Prakash D. (2019). Nutraceuticals from Microbes of Marine Sources. Nutraceuticals-Past, Present and Future.

[169] Loftsson T., Ilievska B., Asgrimsdottir G.M., Ormarsson O.T., Stefansson E. (2016). Fatty acids from marine lipids: Biological activity, formulation and stability. J. Drug Deliv. Sci. Technol..

[170] Ramesh Kumar B., Deviram G., Mathimani T., Duc P.A., Pugazhendhi A. (2019). Microalgae as rich source of polyunsaturated fatty acids. Biocatal. Agric. Biotechnol..

[171] El Shatshat A., Pham A.T., Rao P.P.N. (2019). Interactions of polyunsaturated fatty acids with amyloid peptides Aβ40 and Aβ42. Arch. Biochem. Biophys..

[172] Mallick R., Basak S., Duttaroy A.K. (2019). Docosahexaenoic acid,22:6n-3: Its roles in the structure and function of the brain. Int. J. Dev. Neurosci..

[173] Zhou M.-M., Ding L., Wen M., Che H.-X., Huang J.-Q., Zhang T.-T., Xue C.-H., Mao X.-Z., Wang Y.-M. (2018). Mechanisms of DHA-enriched phospholipids in improving cognitive deficits in aged SAMP8 mice with high-fat diet. J. Nutr. Biochem..

[174] Zhang Y.-P., Brown R.E., Zhang P.-C., Zhao Y.-T., Ju X.-H., Song C. (2018). DHA, EPA and their combination at various ratios differently modulated Aβ25-35-induced neurotoxicity in SH-SY5Y cells. Prostaglandins Leukot. Essent. Fat. Acids.

[175] Wang C.-C., Wang D., Zhang T.-T., Yanagita T., Xue C.-H., Chang Y.-G., Wang Y.-M. (2018). A comparative study about EPA-PL and EPA-EE on ameliorating behavioral deficits in MPTP-induced mice with Parkinson’s disease by suppressing oxidative stress and apoptosis. J. Funct. Foods.

[176] Cheng-Sánchez I., Sarabia F. (2018). Chemistry and Biology of Bioactive Glycolipids of Marine Origin. Mar. Drugs.

[177] Ouellette R.J., Rawn J.D., Ouellette R.J., Rawn J.D. (2018). 31—Lipids and Biological Membranes. Organic Chemistry.

[178] Aerts J.M.F.G., Kuo C.-L., Lelieveld L.T., Boer D.E.C., van der Lienden M.J.C., Overkleeft H.S., Artola M. (2019). Glycosphingolipids and lysosomal storage disorders as illustrated by gaucher disease. Curr. Opin. Chem. Biol..

[179] Vasconcelos A.A., Pomin V.H. (2018). Marine Carbohydrate-Based Compounds with Medicinal Properties. Mar. Drugs.

[180] Li Q., Che H.X., Wang C.C., Zhang L.Y., Ding L., Xue C.H., Zhang T.T., Wang Y.M. (2019). Cerebrosides from Sea Cucumber Improved Aβ 1–42 -Induced Cognitive Deficiency in a Rat Model of Alzheimer’s Disease. Mol. Nutr. Food Res..

[181] Lopez P.H.H., Báez B.B., Schnaar R.L., Lopez P.H.H. (2018). Chapter Thirteen—Gangliosides in Axon Stability and Regeneration. Progress in Molecular Biology and Translational Science.

[182] Wang X., Tao S., Cong P., Wang Y., Xu J., Xue C. (2017). Neuroprotection of Strongylocentrotus nudus gangliosides against Alzheimer’s disease via regulation of neurite loss and mitochondrial apoptosis. J. Funct. Foods.

[183] Zhang J., Li C., Yu G., Guan H. (2014). Total synthesis and structure-activity relationship of glycoglycerolipids from marine organisms. Mar. drugs.

[184] Floris R., Rizzo C., Giudice A.L. (2018). Biosurfactants from Marine Microorganisms. Metabolomics-New Insights into Biology and Medicine.

[185] Sun X.-M., Ren L.-J., Zhao Q.-Y., Ji X.-J., Huang H. (2018). Microalgae for the production of lipid and carotenoids: A review with focus on stress regulation and adaptation. Biotechnol. Biofuels.

[186] Sajjadi B., Chen W.-Y., Raman A.A.A., Ibrahim S. (2018). Microalgae lipid and biomass for biofuel production: A comprehensive review on lipid enhancement strategies and their effects on fatty acid composition. Renew. Sustain. Energy Rev..

[187] Ramesh C., Vinithkumar N.V., Kirubagaran R., Venil C.K., Dufossé L. (2019). Multifaceted applications of microbial pigments: Current knowledge, challenges and future directions for public health implications. Microorganisms.

[188] Ramesh C., Vinithkumar N., Kirubagaran R. (2019). Marine pigmented bacteria: A prospective source of antibacterial compounds. J. Nat. Sci. Biol. Med..

[189] Darwesh O.M., Barakat K.M., Mattar M.Z., Sabae S.Z., Hassan S.H. (2019). Production of antimicrobial blue green pigment pyocyanin by marine Pseudomonas aeruginosa. Biointerface Res. Appl. Chem..

[190] Pathak J., Mondal S., Ahmed H., Rajneesh, Singh S.P., Sinha R.P. (2019). In silico study on interaction between human polo-like kinase 1 and cyanobacterial sheath pigment scytonemin by molecular docking approach. Biointerface Res. Appl. Chem.

[191] Varmira K., Habibi A., Moradi S., Bahramian E. (2018). Experimental Evaluation of Airlift Photobioreactor for Carotenoid Pigments Production by Rhodotorula rubra. Rom. Biotechnol. Lett..

[192] Torregrosa-Crespo J., Montero Z., Fuentes J.L., Reig García-Galbis M., Garbayo I., Vílchez C., Martínez-Espinosa R.M. (2018). Exploring the Valuable Carotenoids for the Large-Scale Production by Marine Microorganisms. Mar. Drugs.

[193] Galasso C., Corinaldesi C., Sansone C. (2017). Carotenoids from Marine Organisms: Biological Functions and Industrial Applications. Antioxidants.

[194] Sathasivam R., Ki J.-S. (2018). A Review of the Biological Activities of Microalgal Carotenoids and Their Potential Use in Healthcare and Cosmetic Industries. Mar. Drugs.

[195] Huang C., Wen C., Yang M., Gan D., Fan C., Li A., Li Q., Zhao J., Zhu L., Lu D. (2019). Lycopene protects against t-BHP-induced neuronal oxidative damage and apoptosis via activation of the PI3K/Akt pathway. Mol. Biol. Rep..

[196] Liu C.B., Wang R., Yi Y.F., Gao Z., Chen Y.Z. (2018). Lycopene mitigates β-amyloid induced inflammatory response and inhibits NF-κB signaling at the choroid plexus in early stages of Alzheimer‘s disease rats. J. Nutr. Biochem..

[197] Cao Z., Wang P., Gao X., Shao B., Zhao S., Li Y. (2019). Lycopene attenuates aluminum-induced hippocampal lesions by inhibiting oxidative stress-mediated inflammation and apoptosis in the rat. J. Inorg. Biochem..

[198] Man H.B., Bi W.P. (2018). Protective effect of lycopene in a mouse model of Parkinson’s disease via reducing oxidative stress and apoptosis. Anal. Quant. Cytopathol. Histopathol..

[199] Hua Y., Xu N., Ma T., Liu Y., Xu H., Lu Y. (2019). Anti-inflammatory effect of lycopene on experimental spinal cord ischemia injury via cyclooxygenase-2 suppression. NeuroImmunoModulation.

[200] Wen S.X., Yang W.C., Shen Z.Y., Wang W., Hu M.Y. (2019). Protective effects of lycopene on cerebral vessels and neurons of hyperlipidemic model rats. Chin. J. Pharmacol. Toxicol..

[201] Han J.H., Lee Y.S., Im J.H., Ham Y.W., Lee H.P., Han S.B., Hong J.T. (2019). Astaxanthin Ameliorates Lipopolysaccharide-Induced Neuroinflammation, Oxidative Stress and Memory Dysfunction through Inactivation of the Signal Transducer and Activator of Transcription 3 Pathway. Mar. Drugs.

[202] Rahman S.O., Panda B.P., Parvez S., Kaundal M., Hussain S., Akhtar M., Najmi A.K. (2019). Neuroprotective role of astaxanthin in hippocampal insulin resistance induced by Aβ peptides in animal model of Alzheimer’s disease. Biomed. Pharmacother..

[203] Lu Y., Wang X., Feng J., Xie T., Si P., Wang W. (2019). Neuroprotective effect of astaxanthin on newborn rats exposed to prenatal maternal seizures. Brain Res. Bull..

[204] Alghazwi M., Smid S., Musgrave I., Zhang W. (2019). In vitro studies of the neuroprotective activities of astaxanthin and fucoxanthin against amyloid beta (Aβ 1-42) toxicity and aggregation. Neurochem. Int..

[205] Rao S.V., Hemalatha P., Yetish S., Muralidhara M., Rajini P.S. (2019). Prophylactic neuroprotective propensity of Crocin, a carotenoid against rotenone induced neurotoxicity in mice: Behavioural and biochemical evidence. Metab. Brain Dis..

[206] Haeri P., Mohammadipour A., Heidari Z., Ebrahimzadeh-bideskan A. (2019). Neuroprotective effect of crocin on substantia nigra in MPTP-induced Parkinson’s disease model of mice. Anat. Sci. Int..

[207] Wang C., Cai X., Hu W., Li Z., Kong F., Chen X., Wang D. (2019). Investigation of the neuroprotective effects of crocin via antioxidant activities in HT22 cells and in mice with Alzheimer’s disease. Int. J. Mol. Med..

[208] Zhou L., Ouyang L., Lin S., Chen S., Liu Y., Zhou W., Wang X. (2018). Protective role of β-carotene against oxidative stress and neuroinflammation in a rat model of spinal cord injury. Int. Immunopharmacol..

